# Preclinical Models of Visceral Sarcomas

**DOI:** 10.3390/biom13111624

**Published:** 2023-11-06

**Authors:** Alice Costa, Livia Gozzellino, Margherita Nannini, Annalisa Astolfi, Maria Abbondanza Pantaleo, Gianandrea Pasquinelli

**Affiliations:** 1 IRCCS Azienda Ospedaliero-Universitaria di Bologna, 40138 Bologna, Italy; alice.costa@aosp.bo.it; 2 Department of Medical and Surgical Sciences (DIMEC), University of Bologna, 40138 Bologna, Italy; 3 Division of Oncology, IRCCS Azienda Ospedaliero-Universitaria di Bologna, 40138 Bologna, Italy; 4 Division of Pathology, IRCCS Azienda Ospedaliero-Universitaria di Bologna, 40138 Bologna, Italy

**Keywords:** soft tissue sarcomas, preclinical models, cancer-derived cells, patient-derived xenografts, genome-engineered mouse models

## Abstract

Visceral sarcomas are a rare malignant subgroup of soft tissue sarcomas (STSs). STSs, accounting for 1% of all adult tumors, are derived from mesenchymal tissues and exhibit a wide heterogeneity. Their rarity and the high number of histotypes hinder the understanding of tumor development mechanisms and negatively influence clinical outcomes and treatment approaches. Although some STSs (~20%) have identifiable genetic markers, as specific mutations or translocations, most are characterized by complex genomic profiles. Thus, identification of new therapeutic targets and development of personalized therapies are urgent clinical needs. Although cell lines are useful for preclinical investigations, more reliable preclinical models are required to develop and test new potential therapies. Here, we provide an overview of the available in vitro and in vivo models of visceral sarcomas, whose gene signatures are still not well characterized, to highlight current challenges and provide insights for future studies.

## 1. Introduction

Visceral sarcomas are rare malignancies belonging to soft tissue sarcomas (STSs). STSs are aggressive tumors which arise from the malignant transformation of mesenchymal cells and comprise very heterogeneous entities, accounting for ~1% of all adult human malignancies [1,2,3]. Visceral sarcomas mainly occur in adults and include several histotypes, including liposarcoma (LPS) and angiosarcoma (AS), as well as some leiomyosarcomas (LMSs) and undifferentiated pleomorphic sarcomas (UPSs). The most common type of visceral sarcoma is the gastrointestinal stromal tumor (GIST), which arises from the muscle wall of the digestive tract [4,5,6]. All these neoplasms are believed to derive from the transformation of a common multipotent cell of origin, which can differentiate into specific lineages [7,8]. The precise mechanisms associated with sarcoma development remain to be clarified because of the rarity of the disease and the large number of subtypes [9,10]. Indeed, STSs comprise over 70 different histotypes, each characterized by a distinct morphology and molecular aberrations, which usually correspond to a specific clinical course and to specific therapeutic approaches [11,12]. Only a small percentage of tumors (~20%) displays distinct diagnostic markers: recurrent genetic alterations, such as specific mutations (e.g., *KIT* activating mutation in gastrointestinal stromal tumors) and chromosomal translocations (observed in approximately 20% of STSs), or complex genomic profiles and mutated tumor suppressor genes, including *TP53* and retinoblastoma 1 (*RB1*) [9,13,14]. However, the majority of STSs have nonspecific genetic alteration with a complex karyotype [15].

Despite their heterogeneity, the first-line treatment for most sarcoma subtypes is still limited to traditional surgery, with or without adjuvant or neoadjuvant radiotherapy, and represents the most common treatment for localized sarcomas. Unfortunately, most patients develop metastasis, which is treated with chemotherapy, principally doxorubicin and ifosfamide, two main active agents reaching a response rate higher than 20% in advanced STSs [16,17]. Nonetheless, the high degree of heterogeneity combined with the poor knowledge of tumor molecular drivers limits treatment efficacy, especially in the case of unresectable, recurrent, or metastatic disease. Moreover, due to the prevalence of many molecular aberrations within specific sarcomas, few STSs are therapeutically targeted [7,9] with a 5-year survival rate of ~50% and <10% for localized and metastatic STSs, respectively [16,18].

Thus, the identification of new genomic and molecular therapeutic targets and preclinical testing of personalized therapies remain urgent unmet medical needs, especially for visceral sarcomas. However, this requires the development of reliable and predictive tumor models to test only the most promising therapies in clinical trials. To date, the most used preclinical models in the field include genetically engineered or transgenic mice, xenografts, and PDXs (patient-derived xenografts) [19]. However, these models present significant limitations, including high management costs, lengthy experimental processes, and the variable adherence of the animal model to human pathology. In this regard, given the rarity of visceral sarcomas, several studies focus on the most common STSs, mainly skeletal sarcomas. Thus, in this review, we aim to provide an overview of the current in vitro and in vivo models developed for visceral sarcomas, to highlight related challenges and offer insights for forthcoming research.

## 2. Advances in In Vitro Models

### 2.1. Established Cancer-Derived Cell Lines

Historically, cell models have been the primary research tool for the study of STSs, providing valuable insights into sarcoma biology, such as tumor progression and metastasis. Nonetheless, cancer cell lines present several limitations. For instance, prolonged cell culture can determine the onset of secondary genetic changes, including copy number variations and transcriptomic drifts, or specific clone selection, leading to biased results. Additionally, the research findings suggest that tumor cell lines are more similar to one other than to the original clinical samples, which explains their limited utility. In fact, the in vitro and in vivo drug response of certain cell lines was not frequently recapitulated in numerous clinical trials [20,21,22].

Many sarcoma cell lines have been established over the years. However, several histological subtypes are largely underrepresented and only a few well-characterized cell lines are publicly available; moreover, many of them are not fully representative of the corresponding tumor tissue [1,8].

Hattori and colleagues investigated the current status of the reported sarcoma cell lines, observing that only 139 out of 819 sarcoma cell lines, named according to the WHO classification, have been deposited into public cell banks. They also observed that among the 189 histotypes listed in the WHO classification, only 45 have corresponding cell lines [23]. Thus, an increased number of cell lines representing numerous sarcoma histological subtypes is necessary to effectively understand the complexity of the disease and enhance the utility of tumor cell cultures in cancer research.

Table 1 shows some examples of the available human cell lines derived from different sarcomas.

In vitro studies with these cell lines have been fundamental in sarcoma research, mainly for drug screening, but also for a better comprehension of the mechanisms behind tumor progression and metastasis [8]. An excellent example of successful translational research is represented by the human GIST cell lines widely employed to predict the role of c-KIT signaling and to evaluate the efficiency of imatinib (IM) as a new targeted therapy. Different *KIT* mutations affect the apoptotic signal transduction caused by imatinib in GISTs, as observed by Noma and colleagues in the GIST-T1 and the GIST882 lines, carrying activating mutant KIT. However, development of imatinib resistance is frequent, and understanding the underlying mechanisms of this event is crucial to establish new treatment strategies [45]. Thus, alternative approaches have been proposed, such as the inhibition of KIT-dependent signaling pathways or their indirect inhibition by using HSP90 inhibitors (HSP90i). Preclinical studies on tumor cell lines have extensively investigated small-molecule HSP90i, which appears to be a promising strategy to overcome imatinib resistance [46]. Some inhibitors are currently undergoing phase II/III clinical trials in cancer patients, either as standalone treatments or in combination with traditional chemotherapy [47]. The effects of new classes of anti-cancer agents involved in epigenetic and non-epigenetic regulation, alone or in combination with other anti-cancer therapies, are widely investigated in tumor cell lines [48]. In this regard, Mühlenberg’s group observed that the histone deacetylase (HDAC) inhibitors (HDACi) SAHA and panobinostat (LBH589) hinder cell growth in KIT-positive (GIST-T1, GIST822, and GIST48) but not in *KIT*-negative cells (GIST62, GIST48B, and GIST522). This proves that HDACi primarily act by targeting the oncogenic KIT and exert their strong antiproliferative and proapoptotic effects in both IM-sensitive and IM-resistant GISTs. The authors also provided preclinical evidence of the disease-specific impact of HDACi on *KIT*-positive GISTs, which has the potential to be translated into therapeutic activity [29]. To overcome IM resistance in GIST patients, other drugs have been tested over the years, such as sorafenib, which displays clinical activity against TKI-resistant GISTs. Heinrich and colleagues tested this compound in a panel of KIT- and PDGFRA-mutant kinases expressed by the transient transfection of the reference cell lines (GIST-T1/829 and IM-resistant GIST-T1). Sorafenib showed a good IC_50_ potency against all IM-resistant mutations, except those involving *KIT* codon 816 and *PDGFRA* codon 842 [43].

Among the available liposarcoma cell lines, LPS141 cells are valid candidates to test new anti-cancer drugs, while T449 and T778 are more suitable to study tumor progression and recurrence, as demonstrated by Stratford‘s team. A correlation between high proliferation rate and in vivo tumor-forming ability was also observed [26]. Simultaneously, cell lines derived from dedifferentiated liposarcomas carrying 12q13-15 region amplification (including *MDM2* and *CDK4* genes) represent an excellent preclinical model for the study of well-differentiated (WDLPS) and dedifferentiated (DDLPS) liposarcoma development and proliferation. The p53 significance in both cancer development and therapy response has aroused interest in terms of the p53–MDM2 interaction as a possible target for small-molecule therapy over the years [24]. MDM2 inhibition results in antitumor activity and increased apoptosis, as demonstrated by Müller and colleagues in an in vitro study conducted on WDLPS (T449 and T778) and DDLPS (FU-DDLS-1) *TP53* wild-type and *MDM2* amplified cell lines, indicating a potential link between treatment response and the mutational status of these genes [24].

Unfortunately, for some visceral sarcoma subtypes, very few cellular models are available, as in the case of AS [1]. Krump-Konvalinkova and Bittinger were able to establish the human endothelial AS-M cell line from a cutaneous angiosarcoma. These cells displayed endothelial characteristics, as CD31 expression, and might be considered a suitable model to investigate human endothelial cells [49].

Several studies have also been conducted to develop three-dimensional culture systems for sarcomas, with the aim of better resembling the surrounding extracellular matrix and overcoming the disadvantages of two-dimensional cell cultures. Maintaining cells in a 2D culture results in morphological changes, leading to altered gene and protein expression and different cell behavior compared to the original tissue. 3D cellular models can overcome these limitations since the architecture of the original tissue and the native cell-cell and cell–extracellular matrix interactions are preserved [7]. 3D cultures are also valuable tools for studying the microenvironment’s influence on cells within a tumor and their response to drug treatment [50]. However, inducing sarcoma cell proliferation in a 3D culture system is more challenging than in a 2D culture. This can be explained by the impact of the microenvironment on sarcoma cell proliferation, including angiogenic capability, and by the presence of distinct cell–matrix adhesion molecules in 3D and 2D models [7,51,52]. As previously discussed, cells of a specific sarcoma subtype can differentiate into multiple lineages in vitro in the presence of certain inducing factors. Thus, it is clear that not only the cell of origin but also the tumor microenvironment plays a crucial role in determining the final tumor phenotype [7]. The molecular biology of sarcomas is poorly understood, especially the interplay among signaling pathways, as well as the epigenetic modifications and regulatory RNA sequences. Patient-derived 3D sarcoma models could be useful to recapitulate the three-dimensional structure of these neoplasms, clarifying the complex crosstalk between tumor cells and their microenvironment, and contributing to personalized medicine development [7,53].

Despite most of the available three-dimensional spheroid cultures and organoids being relevant to the study of skeletal sarcomas, 3D models have also been developed for visceral sarcomas. In this regard, Escudero and colleagues assessed the effect of eribulin (an antitumor agent approved for advanced liposarcoma treatment) on inhibiting the proliferation, migration, and invasion of LPSs and LMSs in 3D spheroid cell cultures. For the first time, they explored erilubin’s action under 3D conditions in LMSs and LPSs, demonstrating an increase in the GI_50_ (growth inhibitory concentration by 50%) values to a nanomolar range compared to in a 2D cellular context [54].

Roohani and colleagues conducted a pilot study on a STS 3D model developed from patient-derived cell cultures (PD3D) of an untreated localized UPS. They assessed a decrease in tumor cell viability at different time points (four and eight days after treatment) using increasing doses of photon and proton radiation. PD3D therefore could be a valuable tool to facilitate translational studies toward individualized subtype-specific radiotherapy in STS patients [55].

Nonetheless, it is still unclear how three-dimensional models will allow better predictability for treatments that target the microenvironment or involve radiation. Furthermore, the available cell lines inadequately represent sarcoma diversity, and primarily encompass the most common groups like osteosarcomas, rhabdomyosarcomas, and leiomyosarcomas while lacking representation of rarer subtypes [7]. Therefore, new cell lines are urgently needed in the field of sarcoma research [1].

### 2.2. Stem Cell-Based Cancer Models

Cancer stem cells (CSCs) in soft tissue sarcomas can be reprogrammed toward a pluripotent state, using similar methods to those applied to normal cells (e.g., induced pluripotent stem cell (iPSC) technology) [14,56,57]. Recently, iPSCs have emerged as a valuable tool for disease modeling, already applied in the field of genetic and oncological disease. This is due to the possibility of replicating a cancer phenotype within the appropriate cell lineage. By generating genome-edited isogenic iPSC lines in correspondence to the oncogenic drivers within a specific tissue, it would be possible to faithfully reproduce the neoplasm in vitro. This could facilitate the study of the targeted treatment susceptibility of various tumor histotypes [58,59].

While targeting CSCs is crucial for all cancer types, in STSs, it is more challenging since these neoplasms are less common and exhibit high cellular and molecular heterogeneity. However, STSs’ unique characteristics offer opportunities for further exploration, such as the identification of common CSC signatures across all STSs, which can be therapeutically targeted [60]. By combining CRISPR-Cas9 technologies with HDR (homology-directed repair) in human embryonic stem cells (hES), Vanoli and colleagues developed an innovative approach to investigating the cell of origin in human cancers without a clear line of differentiation, including sarcomas with chromosomal translocations. They also demonstrated that chromosomal translocations are involved in hES differentiation in human embryonic-stem-derived mesenchymal progenitor cells (hES-MP), clarifying the role of gene fusion in sarcomagenesis under conditional expression [61].

While several stem cell-based models of synovial sarcoma [62] and skeletal sarcomas have been developed, especially for the study of osteosarcoma [63,64,65] and Ewing’s sarcoma [66], visceral sarcomas need to be further explored.

## 3. Advances in Animal Models

### 3.1. Cell-Derived and Patient-Derived Xenograft Models of Soft Tissue Sarcomas

**Figure 1:**
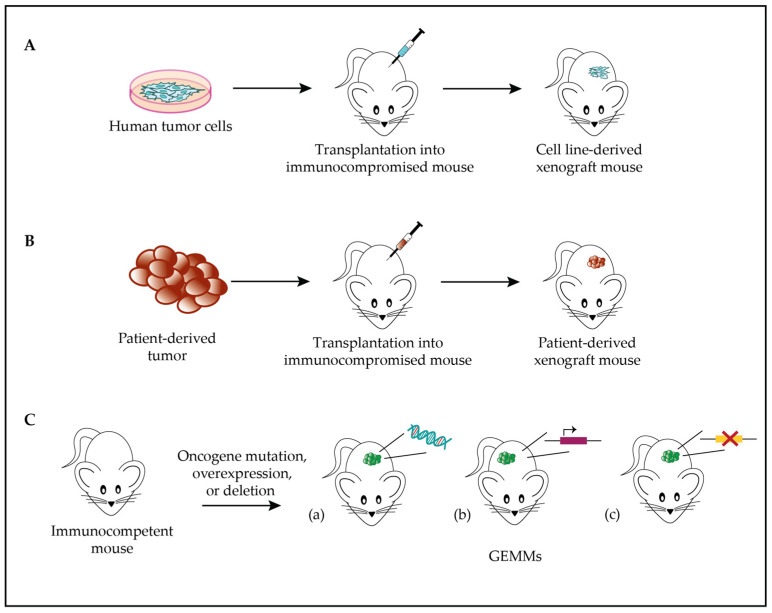
STS models. (**A**). Scheme of cell line-derived xenograft model generation: tumor cells are transplanted into immunocompromised mice to study tumor biology and behavior. (**B**). Scheme of PDX model generation: patient-derived tumor cells are transplanted into immunocompromised mice to study tumor biology and behavior. (**C**). Genome-engineered mouse model: GEMMs drive tumor development via mutation (**a**), overexpression (**b**), or deletion (**c**) of one or several oncogenes.

Among the available animal models in cancer research, human tumor xenografts are the most widely used. They consist of the implantation of tumor cells into immunocompromised mice, either under the skin or into the same organ type of origin (Figure 1A). Athymic nude mice, severe combined immunodeficient (SCID) mice, or other immunocompromised mice can readily accept xenografts [67].

Several studies have been conducted on cell-derived xenograft (CDX) models to assess both the target therapy effectiveness and oncogenic activity in STSs. An example is the xenograft model investigated by Floris and colleagues, obtained from the IM-sensitive GIST882 cell line. For the first time, they used this nude xenograft model to evaluate the effects of the HDACi panobinostat on human GISTs with different oncogenic KIT mutations. Panobinostat reduced proliferation and increased apoptosis in all xenografts, proving its anti-tumor activity [68]. Another STS xenograft model, applied to leiomyosarcomas, is the one established by Zhang‘s group by injecting SCID mice with SK-LMS-1 cells previously transfected with VEGF_165_ [69]. Approximately 21–25% of patients affected by STSs display VEGF overexpression, which correlates with a more advanced tumor grade and a worse prognosis [70,71]. Based on the established role of VEGF and its receptor VEGFR in angiogenesis, the authors tried to better clarify the role of VEGF_165_ in STS growth, metastasis, and chemoresistance. The obtained VEGR_165_-overexpressing xenograft model revealed the significant impact of VEGF expression on STSs’ ability to grow and metastasize, and the anti-VEGFR2 monoclonal antibody’s effects on enhancing the doxorubicin response [69]. CDX models provide the advantage of replicating human tumor biology, despite the possibility of clone selection, which might not accurately represent the human disease [1].

An alternative to cell line xenografts is the transplantation of small pieces of human-derived tumor samples into mice, obtaining so-called patient-derived xenograft (PDX) models (Figure 1B). In this case, the advantage is represented by the close resemblance of the model to the primary tumor sample [72]. For instance, copy number alterations observed in STS PDXs are also detected in sarcoma patients, suggesting that these alterations correlate with actual tumor progression rather than experimental model alterations [73]. Several models have been successfully established, achieving an overall engraftment success rate of 32% to 69% and efficiently reproducing the genetic and phenotypic characteristics of the original tumor [19,74].

PDXs can be considered superior to classical cell-line-derived xenografts in accurately predicting patient response to therapy [75,76]. In this regard, Gebreyohannes and colleagues proved the anti-tumor efficacy of cabozantinib, a novel tyrosine kinase inhibitor, by reducing tumor growth, proliferation, and angiogenesis in IM-sensitive and IM-resistant PDX models of GISTs [77]. Similarly, Van Looy’s team demonstrated the effectiveness of three PI3K inhibitors combined with imatinib in reducing tumor volumes and enhancing apoptosis in GIST PDXs [78]. Analogous research has been conducted on sarcoma xenograft models to explore the antitumor effects of target therapies. This is the case for Li and colleagues, who established two PDX models of DDLPS by implanting pieces of patient-derived sarcomas inro athymic nude NMRI mice. Significantly decreased proliferation and angiogenesis inhibition characterized all the xenografts treated with the TKRi pazopanib [79]. Zuco’s group conducted a direct comparison between the first-in-class XPO1 inhibitor selinexor and doxorubicin, the standard front-line therapy for sarcomas, in three DDLPS PDXs. They demonstrated selinexor’s potential as a therapeutic agent for DDLPS, given its superiority in terms of the umor response in all PDXs when compared to doxorubicin, regardless of the *MDM2* amplification and histological differentiations [80].

The few preclinical models of leiomyosarcoma and the lack of fidelity of the established LMS cell lines to their mesenchymal neoplasm of origin limit the translational understanding of the disease. In this regard, Hemming and colleagues characterized LMS PDX models assessing that, across multiple xenograft passages, the parental tumor histological appearance, copy number variation, and transcriptional program were maintained. Additionally, LMS PDXs were susceptible to cyclin-dependent kinase (CDK) inhibition, which alters the oncogenic transcriptional program driven by E2F and hinders tumor growth. Thus, CDK inhibitors can be a valuable therapeutic strategy for patients with LMS [81].

As previously discussed, PDXs are useful to assess the effects of combined therapies. This was further supported by Perez’s team, who demonstrated the potential therapeutic strategies of doxorubicin and olaparib against sarcomas in UPS PDXs. The combined treatment efficacy in tumors with high levels of pH2AX and MAP17 suggested that both biomarkers could potentially identify patients who would benefit more from the therapy [82]. Similarly, Stacchioti’s group investigated the preclinical antitumor activity of EPZ-011989, an EZH2 inhibitor, in INI1-deficient proximal-type epithelial sarcoma (ES) PDXs. EPZ-011989, gemcitabine, and a doxorubicin–ifosfamide combination exhibited comparable antitumor activity in treated mice, supporting their clinical use as effective therapies. Moreover, EZH2 was confirmed as a viable therapeutic target in ESs, suggesting autophagy as a possible protective mechanism against EZH2 inhibition [83].

Interestingly, an established patient-derived orthotopic nude mouse xenograft of a retroperitoneal STS was developed by Hiroshima and colleagues. This model recapitulated the histology of the original tumor better than the same subcutaneous ectopic model [84]. Hence, PDXs are largely used for the assessment of human tumor biology and have a broad range of applications in preclinical drug testing, in therapeutic target identification, and for the establishment of stable xenograft cell lines [1,85]. Furthermore, these models could represent an option for personalized medicine strategies, allowing for direct testing of potential drug treatments on a model matching the patient [1]. However, recent advances in immunotherapy highlighted the importance of immune response in cancer progression and treatment, and thus the need to develop new PDX models to investigate human cancer and immune system interactions [85]. Wang’s team established an in vivo humanized mouse xenograft model by transplanting human CD34+ hematopoietic progenitor and stem cells into NGS mice, originating humanized NGS (HuNGS) mice with human hematopoietic and immune systems. Then, they implanted PDXs of various cancers (sarcoma, non-small cell lung cancer, bladder, triple-negative breast cancer) into HuNGSs. Treatment with PD-1 checkpoint inhibitor pemrolizumab significantly inhibited the tumor growth of PDX tumors in HuNGS mice, assessing the potential utility of the model for preclinical immunotherapy research [86].

Patient-derived models (PDMs) are widely applied in cancer research, drug development, and clinical applications. Since PDMs are directly derived from patients, they can predict treatment response and could help to identify the best personalized treatment strategy [75,87]. Research findings support that sarcoma patient-derived cells predict STS patient response to therapy since these models preserve the genetic characteristics of the original tumor and the association between drug sensitivity and patient response [88]. Nonetheless, there are still challenges and limitations to overcome, such as costs and time, as well as tumor heterogeneity, which might not be represented by PDMs, potentially affecting the accuracy of drug testing [75,87].

### 3.2. Genome-Engineered Mouse Models of Soft Tissue Sarcomas

Among other animal models used in sarcoma research, it is worthwhile to mention the genome-engineered mouse model (GEMM), in which mice display an altered genetic profile by mutating, deleting, or overexpressing one or several oncogenes (Figure 1C). GEMMs allow us to monitor the effects of induced genetic alterations over time and to evaluate tumor response to treatment in vivo [89]. Conditional transgenic mice expressing oncogenic human fusion genes, as well as immunodeficient mice that enable the growth of human tumor cells or tumor fragments cultured in vitro, have allowed the implementation of the available preclinical models for translational research [90].

An example of an in vivo visceral sarcoma model is represented by the MMTV-CR-1 transgenic mice generated by Strizzi and colleagues, in which *CR-1* transgene expression is regulated by the MMTV (the mouse mammary tumor virus) long terminal repeat promoter and leads to uterine leiomyosarcoma development. CR-1 plays a role in uterine tumor onset via the direct activation of c-src and Akt or via crosstalk with the canonical Wnt signaling pathway [91].

Many transgenic mouse models have also been generated to study gastrointestinal stromal tumors. For instance, Sommer’s group created a knock-in mouse with an exon 11 *KIT*-activating mutation (KITV558 deletion), previously found in the case of human familial GIST syndrome. Through this model, they reproduced gastrointestinal pathology in mice with remarkable penetrance, demonstrating that the constitutive activation of KIT signaling is pivotal and sufficient to cause neoplastic growth in mice [92]. Likewise, Rubin’s team developed a homozygous knock-in mouse model harboring a *KIT*-activating mutation K641E, resulting in GIST development with 100% penetrance. They also showed the model effectiveness for the study of KIT pathway activation, GIST pathogenesis, and preclinical validation of GIST therapies and drug response [93].

Regarding the study of undifferentiated pleomorphic sarcoma, for the first time, Buchakjian and colleagues generated a viral Cre-mediated TRP53/PTEN mouse model, by injecting adenoviral Cre recombinase into *TRP53*flox/flox/*PTEN*flox/flox lox–stop–lox luciferase mice. All the injected mice developed STSs, identified for 93% as invasive pleomorphic sarcomas characterized by lymphocytic infiltrate (64%) and PD-L1 expression (71%). The model could represent a valuable tool for liposarcoma preclinical studies since the homozygous loss of *TRP53* and *PTEN* in mouse adipose tissue also characterizes this sarcoma histotype [94].

Within the realm of liposarcoma research, Pèrez-Mancera’s group generated *CHOP* and *FUS* ± *CHOP* transgenic mice, by introducing *CHOP* or the *FUS-CHOP* transgene into mouse genomes. Interestingly, only the latter mouse group developed LPSs. This demonstrated the critical role of the FUS domain in the liposarcoma pathogenesis and pioneeringly proved the in vivo correlation between gene fusions and solid tumor onset [95]. A few years later, the same group generated double-transgenic *FUS-CHOP* mice to investigate the significance of the FUS-CHOP interaction. As a result, *FUS* expression in *CHOP* transgenic mice restored liposarcoma development, indicating that the FUS and CHOP domains cooperate in mutual liposarcoma restoration [96].

As previously mentioned, unfortunately, angiosarcomas lack valid clinical models that allow new therapy development. However, Salter’s team generated an autochthonous AS mouse model driven by p53 deregulation in VE-cadherin-expressing endothelial cells, using Cdh5-Cre mice. AS arose in mice with a penetrance of 100% upon homozygous deletion of *TRP53*. The re-implantation of AS fragments from Cdh5-Cre, Trp53^fl/fl^ mice yielded a reliable and rapid angiosarcoma model. Moreover, transferring tumor fragments within mice allowed them to establish a novel AS model suitable for preclinical studies and for new therapy development [97].

## 4. Discussion

**Figure 2:**
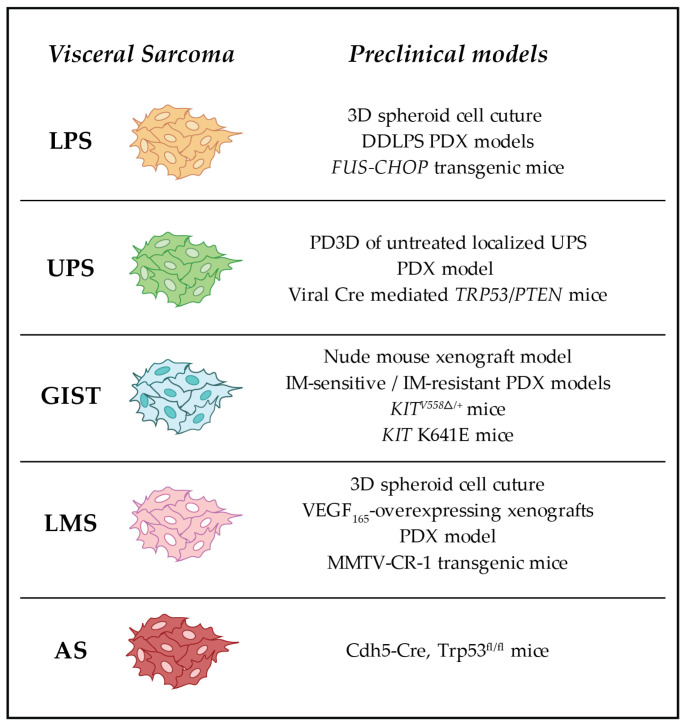
Preclinical models of visceral sarcomas. Schematic representation of the preclinical models of visceral sarcomas discussed in this review. (LPS: liposarcoma; UPS: undifferentiated pleomorphic sarcoma; GIST: gastrointestinal stromal tumor; LMS: leiomyosarcoma; AS: angiosarcoma) [54,55,68,69,77,79,80,81,83,91,92,93,94,95,97].

In this review, we summarize the most used in vitro and in vivo models adopted for the study of visceral sarcomas (Figure 2). However, all of them present both advantages and limitations. Sarcoma cell lines have a central role in cancer research, as they can be easily employed to develop new potential drugs, allowing rapid identification of compounds with anti-cancer activity. Moreover, cell lines can be genetically manipulated to study the effects of specific mutations on tumor growth and invasiveness, and to investigate the molecular mechanisms of visceral sarcomas. Nonetheless, not all the therapeutic approaches can be tested in vitro. Additionally, cell lines might not accurately represent tumors’ microenvironment complexity and their interactions with stromal and immune cells. Thus, the immune system and the effects of drugs targeting the microenvironment cannot be evaluated in cultured cell lines [1,72]. Furthermore, in vitro studies cannot fully recapitulate the genetic and epigenetic landscape of primary tumors. Finally, when maintained in cultures, cells display gene expression and cellular behavior changes over time, which might lead to biased results.

Therefore, in vivo models can compensate for in vitro weaknesses. In particular, xenografts, such as mice injected with subcutaneous cell lines or PDXs, are commonly employed for STS studies. For this purpose, immunocompromised mice are the most used models, although they might select for clones that do not fully represent the human neoplasm. Xenografts have the advantage of allowing rapid compound efficacy screening and precise in vitro cell manipulation (e.g., gene overexpression or knock-down). They can also produce a large number of tumors which can be isolated and analyzed. Nonetheless, the most relevant drawback of xenografts is the lack of a reliable model of human tumor cell and mouse stroma interactions. Another major issue is the fact that the animals are immunocompromised, hindering the immune system’s role in tumor growth, progression, metastasis, and response to therapy [1,8]. On the contrary, in a genetically engineered mouse, tumors arise in an authentic cancer microenvironment and, more importantly, the animal’s immune system remains intact [98]. Thus, compared to xenografts, GEMMs allow for a better replication of the interactions between tumor cells and the host microenvironment, allowing us to test mutation effects in a physiological context. However, to generate and maintain GEM mice is expensive which, added to the complexity of results interpretation, leads to critical limitations [89].

In the field of sarcoma research, non-murine models, such as genetically modified zebrafish, have also been developed, but they primarily concern rhabdomyosarcomas, Ewing’s sarcoma, malignant peripheral nerve sheath tumors, chordomas [8,99], and, to the best of our knowledge, only one liposarcoma case [100].

Visceral sarcomas are generally investigated after the completion of the transformation process. Hence, there is a compelling need to establish robust preclinical models that can faithfully replicate sarcomagenesis in vitro and in vivo. Simultaneously, the need to discover new potential targets for personalized therapies is increasingly relevant. This requires the establishment of reliable and predictive sarcoma models of patient response, to test only the most promising therapeutic approaches in clinical trials. Future research should define the specific phenotype of cancer cells at the origin of different sarcoma histotypes and elucidate the mechanisms driving their transformation. In this regard, a valuable research tool is represented by induced pluripotent stem cells. This model has already been effectively applied in the field of genetic and oncological diseases, thanks to its capability to reproduce disease phenotypes within the appropriate cellular lineage [57]. The ability to derive tissue-specific isogenic iPSC lines, modified via genome editing at the level of driver oncogenes, would allow us to mimic different sarcoma subtypes in vitro. iPSCs, along with three-dimensional cellular systems, might overcome the issues associated with the use of animal models and the limitations of the 2D cultures discussed in this review.

## Figures and Tables

**Table 1:** Examples of available visceral sarcoma cell lines.

Tumor Type	Cell Line	Reference
Liposarcoma (LPS)	FU-DDLS-1	[24,25,26]
	SW872	[24,25,26,27,28], ATCC HTB 92
	T778	[24,25,26,28]
	T449	[24,25,26,28]
	T1000	[25,26]
	LPS141	[3,25,26,29,30,31,32,33,34]
	LP6	[25,35,36]
	GOT3	[25,26,37]
	LISA-2	[25,26,28,38]
Leiomyosarcoma (LMS)	SK-LMS-1	[3], ATCC HTB 88
	SK-UT-1B	[3]
	SK-UT-1	[3], ATCC HTB 114
Gastrointestinal stromal tumor (GIST)	GIST-T1	[29,39,40,41,42,43,44]
	GIST882	[29,39,40,45,46]
	GIST48	[29,40,43,45]
	GIST430	[40,43,44,45,46]
	GIST62	[29]
ATCC, American Type Culture Collection		

## Data Availability

Not applicable.
